# Three-dimensional hydrogen-bonded supra­molecular assembly in tetrakis­(1,3,5-triaza-7-phosphaadamantane)copper(I) chloride hexa­hydrate

**DOI:** 10.1107/S1600536808008179

**Published:** 2008-04-02

**Authors:** Alexander M. Kirillov, Piotr Smoleński, M. Fátima C. Guedes da Silva, Maximilian N. Kopylovich, Armando J. L. Pombeiro

**Affiliations:** a Centro de Química Estrutural, Complexo Interdisciplinar, Instituto Superior Técnico, TU Lisbon, Av. Rovisco Pais, 1049-001 Lisbon, Portugal; b Universidade Lusófona de Humanidades e Tecnologias, ULHT Lisbon, Av. do Campo Grande 376, 1749-024 Lisbon, Portugal

## Abstract

The structure of the title compound, [Cu(PTA)_4_]Cl·6H_2_O (PTA is 1,3,5-triaza-7-phosphaadamantane, C_6_H_12_N_3_P), is composed of discrete monomeric [Cu(PTA)_4_]^+^ cations, chloride anions and uncoordinated water mol­ecules. The Cu^I^ atom exhibits tetra­hedral coordination geometry, involving four symmetry-equivalent P–bound PTA ligands. The structure is extended to a regular three-dimensional supra­molecular framework *via* numerous equivalent O—H⋯N hydrogen bonds between all solvent water mol­ecules (six per cation) and all PTA N atoms, thus simultaneously bridging each [Cu(PTA)_4_]^+^ cation with 12 neighbouring units in multiple directions. The study also shows that PTA can be a convenient ligand in crystal engineering for the construction of supra­molecular architectures.

## Related literature

For general background, see: Kirillov *et al.* (2007 ▶, 2008 ▶); Karabach *et al.* (2006 ▶); Di Nicola *et al.* (2007 ▶). For a comprehensive review of PTA chemistry, see: Phillips *et al.* (2004 ▶). For PTA-derived polymeric networks, see: Lidrissi *et al.* (2005 ▶); Frost *et al.* (2006 ▶); Mohr *et al.* (2006 ▶). For related compounds, see: Forward *et al.* (1996 ▶); Darensbourg *et al.* (1997 ▶, 1999 ▶).
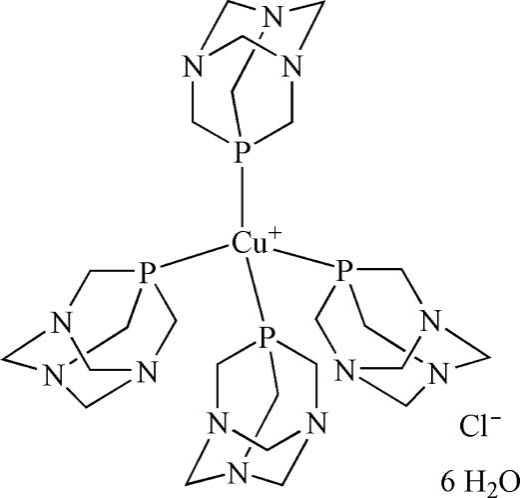


## Experimental

### 

#### Crystal data

[Cu(C_6_H_12_N_3_P)_4_]Cl·6H_2_O
*M* *_r_* = 835.71
Cubic, 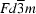

*a* = 19.795 (4) Å
*V* = 7757 (3) Å^3^
*Z* = 8
Mo *K*α radiation
μ = 0.85 mm^−1^
*T* = 150 (2) K
0.20 × 0.17 × 0.12 mm

#### Data collection

Bruker APEXII CCD area-detector diffractometer
Absorption correction: multi-scan (*SADABS*; Sheldrick, 2003 ▶) *T*_min_ = 0.848, *T*_max_ = 0.905
3022 measured reflections
447 independent reflections
361 reflections with *I* > 2σ(*I*)
*R*_int_ = 0.049

#### Refinement

*R*[*F* ^2^ > 2σ(*F* ^2^)] = 0.034
*wR*(*F* ^2^) = 0.092
*S* = 1.08
447 reflections
28 parameters
H-atom parameters constrained
Δρ_max_ = 0.75 e Å^−3^
Δρ_min_ = −0.32 e Å^−3^

### 

Data collection: *APEX2* (Bruker, 2004 ▶); cell refinement: *SAINT* (Bruker, 2004 ▶); data reduction: *SAINT*; program(s) used to solve structure: *SIR97* (Altomare *et al.*, 1999 ▶); program(s) used to refine structure: *SHELXL97* (Sheldrick, 2008 ▶); molecular graphics: *ORTEPIII* (Burnett & Johnson, 1996 ▶), *PLATON* (Spek, 2003 ▶) and *Mercury* (Macrae *et al.*, 2006 ▶); software used to prepare material for publication: *SHELXL97*.

## Supplementary Material

Crystal structure: contains datablocks I. DOI: 10.1107/S1600536808008179/dn2329sup1.cif

Structure factors: contains datablocks I. DOI: 10.1107/S1600536808008179/dn2329Isup2.hkl

Additional supplementary materials:  crystallographic information; 3D view; checkCIF report

## Figures and Tables

**Table 1:** Hydrogen-bond geometry (Å, °)

*D*—H⋯*A*	*D*—H	H⋯*A*	*D*⋯*A*	*D*—H⋯*A*
O10—H10⋯N1	0.81	2.04	2.843 (3)	174

**Fig. 1.**
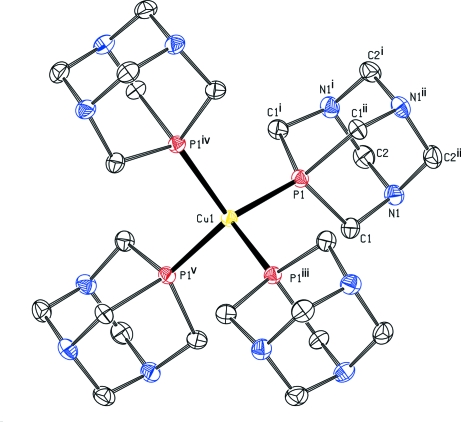
Molecular view of the cation with the atom-labelling scheme. Displacement ellipsoids are drawn at the 50% probability level. Hydrogen atoms are omitted for clarity. [Symmetry codes: (i) z, x, y; (ii) y, z, x; (iii) -x+1/4, y, -z+1/4; (iv) -x+1/4, -y+1/4, z; (v) x, -y+1/4, -z+1/4]

**Fig. 2.**
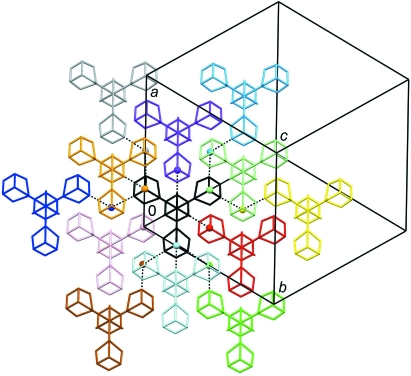
Fragment of the crystal packing diagram of (I) showing the simultaneous multidimensional interlinkage of the central monomeric [Cu(PTA)4]+ unit (black coloured) with twelve neighbouring ones (each represented by different colour) via repeating O10—H10···N1 hydrogen bonding interactions (black dashed lines) between crystallization water molecules O10 (coloured balls) and PTA N1 atoms. H and Cl atoms are omitted for clarity.

**Fig. 3.**
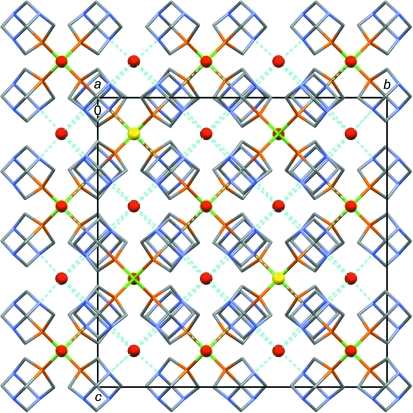
Fragment of the crystal packing diagram of (I) (view along the a axis) showing the extensive hydrogen bonding interactions (pale blue dashed lines) resulting in the formation of a regular three-dimensional H-bonded supramolecular assembly. H atoms are omitted for clarity. Cu, green; P, orange; N, blue; C, grey; O, red (balls); Cl, yellow (balls).

## supplementary crystallographic information

### Comment

1,3,5-triaza-7-phosphaadamantane (PTA) is a water soluble aminophosphine that has sparked recent interest in coordination chemistry in view of the significance of transition metal PTA complexes in aqueous phase catalysis, photochemistry and medicinal chemistry (Phillips *et al.*, 2004). Besides, PTA and its derivatives can also be convenient building blocks for the construction of polymeric networks (Lidrissi *et al.*, 2005; Frost *et al.*, 2006; Mohr *et al.*, 2006) due to several potentially available coordination sites, protonation ability of N atoms, and strong affinity towards hydrogen bonds. Nevertheless, the use of PTA ligands in crystal design and engineering has remained little explored. Hence, in pursuit of our recent studies directed towards the synthesis of new copper compounds including PTA complexes (Kirillov *et al.*, 2007) and various coordination polymers, supramolecular frameworks and host–guest systems with other ligands (Karabach *et al.*, 2006; Di Nicola *et al.*, 2007; Kirillov *et al.*, 2008), we have prepared compound (I) whose crystal structure and supramolecular features are reported herein.

The moiety formula of (I) consists of the [Cu(PTA)_4_]^+^ cation (Fig. 1), one chloride anion and six symmetry equivalent crystallization water molecules. The [Cu(PTA)_4_]^+^ unit possesses a very high symmetry, being generated from only five symmetry nonequivalent atoms (Cu1, P1, N1, C1 and C2). The Cu^I^ atom lies on -43*m* site symmetry and its coordination environment is filled by four equivalent *P*–bound PTA ligands, arranged in a perfect tetrahedral coordination geometry with the corresponding P—Cu—P angles of 109.47 (2)°. The Cu—P bond distances of 2.2598 (6) Å as well as other bonding parameters within the cage-like PTA cores are comparable to those reported for tetrahedral PTA complexes of Cu (Kirillov *et al.*, 2007), Au (Forward *et al.*, 1996), Pt (Darensbourg *et al.*, 1999) and Ni (Darensbourg *et al.*, 1997).

An interesting feature of (I) consists in the extensive intermolecular hydrogen bonding that arises from only one type of O-H···N H-bond (Table 1). Hence, each crystallization water molecule (O10) repeatedly acts as a double H-bond donor bridging to two N1 atoms of two different [Cu(PTA)_4_]^+^ units. This results in the extensive interlinkage in multiple directions of every monomeric copper unit with twelve neighbouring ones (Fig. 2), thus leading to the formation of a regular three-dimensional supramolecular framework (Fig. 3). That framework has the shortest Cu···Cu separation of 13.977 (1) Å and possesses the repeating channels (*ca* 4.8 Å diameter) filled by water molecules.

### Experimental

To the ethanolic solution (5 ml) of CuCl_2_ (27 mg, 0.20 mmol) was added solid PTA (126 mg, 0.80 mmol). The obtained mixture was refluxed for 3 h resulting in a white suspension. This was filtered off and the colourless filtrate was left to evaporate in a beaker in air and at ambient temperature. A small crop of the colourless X-ray quality crystals of (I) was formed in several days. ^1^H NMR data are similar to those reported for [Cu(PTA)_4_]NO_3_ (Kirillov *et al.*, 2007). FT–IR (KBr pellet), cm^-1^: 3430 m, br and 3195 w [ν(H_2_O)], 2940 m and 2901 m [ν_as_(C—H)], 2863 m and 2808 w [ν_s_(C—H)], 1645 w br [δ(H_2_O)], 1437 m, 1413 m, 1365 m, 1296 s, 1242 s, 1180 m, 1105 m, 1037 w, 1015 s, 971 s, 906 w, 890 m, 808 s, 797 s, 744 m, 694 m, 670 w, 582 s, 551 w, 451 s, 406 m [PTA bands]. FAB-MS^+^ (*m*-nitrobenzylalcohol), *m*/*z*: 691 [Cu(PTA)_4_]^+^.

### Refinement

All H atoms attached to C atoms were fixed geometrically and treated as riding with C—H = 0.97 Å and U_iso_(H) = 1.2U_eq_(C). H atom of the water molecule were located in difference Fourier maps and included in the subsequent refinement using restraint (O-H= 0.82 (1)Å) with U_iso_(H) = 1.5U_eq_(O). In the last stage of refinement,it was treated as riding on the O atom.

### Crystal data

**Table:**

[Cu(C_6_H_12_N_3_P)_4_]Cl·6H_2_O	*Z* = 8
*M**_r_* = 835.71	*F*_000_ = 3536
Cubic, *F**d*3*m*	*D*_x_ = 1.431 Mg m^−^^3^
Hall symbol: -F 4vw 2vw 3	Mo *K*α radiation λ = 0.71069 Å
*a* = 19.795 (4) Å	Cell parameters from 743 reflections
*b* = 19.795 (4) Å	θ = 2.9–27.0º
*c* = 19.795 (4) Å	µ = 0.85 mm^−^^1^
α = 90º	*T* = 150 (2) K
β = 90º	Prism, colourless
γ = 90º	0.20 × 0.17 × 0.12 mm
*V* = 7757 (3) Å^3^	

### Data collection

**Table:**

Bruker APEXII CCD area-detector diffractometer	447 independent reflections
Radiation source: fine-focus sealed tube	361 reflections with *I* > 2σ(*I*)
Monochromator: graphite	*R*_int_ = 0.049
*T* = 150(2) K	θ_max_ = 27.0º
φ and ω scans	θ_min_ = 2.9º
Absorption correction: multi-scan(SADABS; Sheldrick, 2003)	*h* = −24→23
*T*_min_ = 0.848, *T*_max_ = 0.905	*k* = −16→11
3022 measured reflections	*l* = −6→25

### Refinement

**Table:**

Refinement on *F*^2^	Secondary atom site location: difference Fourier map
Least-squares matrix: full	Hydrogen site location: inferred from neighbouring sites
*R*[*F*^2^ > 2σ(*F*^2^)] = 0.034	H-atom parameters constrained
*wR*(*F*^2^) = 0.092	*w* = 1/[σ^2^(*F*_o_^2^) + (0.0463*P*)^2^ + 19.2954*P*] where *P* = (*F*_o_^2^ + 2*F*_c_^2^)/3
*S* = 1.08	(Δ/σ)_max_ < 0.001
447 reflections	Δρ_max_ = 0.75 e Å^−^^3^
28 parameters	Δρ_min_ = −0.32 e Å^−^^3^
Primary atom site location: structure-invariant direct methods	Extinction correction: none

### Special details

**Table:**

Geometry. All esds (except the esd in the dihedral angle between two l.s. planes) are estimated using the full covariance matrix. The cell esds are taken into account individually in the estimation of esds in distances, angles and torsion angles; correlations between esds in cell parameters are only used when they are defined by crystal symmetry. An approximate (isotropic) treatment of cell esds is used for estimating esds involving l.s. planes.
Refinement. Refinement of *F*^2^ against ALL reflections. The weighted *R*-factor *wR* and goodness of fit *S* are based on *F*^2^, conventional *R*-factors *R* are based on *F*, with *F* set to zero for negative *F*^2^. The threshold expression of *F*^2^ > σ(*F*^2^) is used only for calculating *R*-factors(gt) *etc*. and is not relevant to the choice of reflections for refinement. *R*-factors based on *F*^2^ are statistically about twice as large as those based on *F*, and *R*- factors based on ALL data will be even larger.

### Fractional atomic coordinates and isotropic or equivalent isotropic displacement parameters (Å^2^)

**Table:**

	*x*	*y*	*z*	*U*_iso_*/*U*_eq_	Occ. (<1)
C1	0.25075 (10)	0.15137 (15)	0.25075 (10)	0.0199 (6)	
H1A	0.2258	0.1228	0.2818	0.024*	0.50
H1B	0.2818	0.1228	0.2258	0.024*	0.50
C2	0.33080 (15)	0.24509 (11)	0.24509 (11)	0.0239 (7)	
H2A	0.3607	0.2726	0.2726	0.029*	
H2B	0.3587	0.2166	0.2166	0.029*	
N1	0.29002 (8)	0.20160 (12)	0.29002 (8)	0.0212 (6)	
Cu1	0.1250	0.1250	0.1250	0.0134 (3)	
P1	0.19090 (4)	0.19090 (4)	0.19090 (4)	0.0156 (3)	
Cl1	0.3750	0.3750	0.3750	0.0165 (5)	
O10	0.3750	0.12300 (14)	0.3750	0.0240 (7)	
H10	0.3521	0.1480	0.3521	0.036*	

### Atomic displacement parameters (Å^2^)

**Table:**

	*U*^11^	*U*^22^	*U*^33^	*U*^12^	*U*^13^	*U*^23^
C1	0.0193 (8)	0.0212 (14)	0.0193 (8)	−0.0005 (7)	−0.0053 (11)	−0.0005 (7)
C2	0.0193 (15)	0.0262 (10)	0.0262 (10)	−0.0036 (8)	−0.0036 (8)	−0.0027 (12)
N1	0.0218 (8)	0.0202 (13)	0.0218 (8)	−0.0019 (7)	−0.0056 (10)	−0.0019 (7)
Cu1	0.0134 (3)	0.0134 (3)	0.0134 (3)	0.000	0.000	0.000
P1	0.0156 (3)	0.0156 (3)	0.0156 (3)	−0.0009 (3)	−0.0009 (3)	−0.0009 (3)
Cl1	0.0165 (5)	0.0165 (5)	0.0165 (5)	0.000	0.000	0.000
O10	0.0255 (10)	0.0210 (16)	0.0255 (10)	0.000	−0.0083 (12)	0.000

### Geometric parameters (Å, °)

**Table:**

C1—N1	1.482 (3)	C2—H2A	0.9700
C1—P1	1.849 (3)	C2—H2B	0.9700
C1—H1A	0.9700	Cu1—P1	2.2596 (13)
C1—H1B	0.9700	P1—C1^i^	1.849 (3)
C2—N1^i^	1.478 (2)	O10—H10	0.8104
C2—N1	1.478 (2)		
			
N1—C1—P1	112.8 (2)	P1—Cu1—P1^iv^	109.5
N1—C1—H1A	109.0	P1^iii^—Cu1—P1^iv^	109.5
P1—C1—H1B	109.0	P1—Cu1—P1^v^	109.5
H1A—C1—H1B	107.8	P1^iii^—Cu1—P1^v^	109.5
N1^i^—C2—N1	113.7 (3)	P1^iv^—Cu1—P1^v^	109.5
N1—C2—H2A	108.8	C1^ii^—P1—C1^i^	97.57 (12)
N1—C2—H2B	108.8	C1^ii^—P1—C1	97.57 (12)
H2A—C2—H2B	107.7	C1^i^—P1—C1	97.57 (12)
C2^ii^—N1—C2	108.5 (3)	C1^ii^—P1—Cu1	119.70 (9)
C2^ii^—N1—C1	111.21 (16)	C1^i^—P1—Cu1	119.70 (9)
C2—N1—C1	111.21 (16)	C1—P1—Cu1	119.70 (9)
P1—Cu1—P1^iii^	109.5		

Symmetry codes: (i) *z*, *x*, *y*; (ii) *y*, *z*, *x*; (iii) −*x*+1/4, *y*, −*z*+1/4; (iv) −*x*+1/4, −*y*+1/4, *z*; (v) *x*, −*y*+1/4, −*z*+1/4.

### Hydrogen-bond geometry (Å, °)

**Table:**

*D*—H···*A*	*D*—H	H···*A*	*D*···*A*	*D*—H···*A*
O10—H10···N1	0.81	2.04	2.843 (3)	174

## References

[bb1] Altomare, A., Burla, M. C., Camalli, M., Cascarano, G. L., Giacovazzo, C., Guagliardi, A., Moliterni, A. G. G., Polidori, G. & Spagna, R. (1999). *J. Appl. Cryst.* **32**, 115–119.

[bb2] Bruker (2004). *APEX2* and *SAINT* Bruker AXS Inc., Madison, Wisconsin, USA.

[bb3] Burnett, M. N. & Johnson, C. K. (1996). *ORTEPIII.* Report ORNL-6895. Oak Ridge National Laboratory, Tennessee, USA.

[bb4] Darensbourg, D. J., Decuir, T. J., Stafford, N. W., Robertson, J. B., Draper, J. D., Reibenspies, J. H., Katho, A. & Joo, F. (1997). *Inorg. Chem.* **36**, 4218–4226.

[bb5] Darensbourg, D. J., Robertson, J. B., Larkins, D. L. & Reibenspies, J. H. (1999). *Inorg. Chem.* **38**, 2473–2481.

[bb6] Di Nicola, C., Karabach, Y. Y., Kirillov, A. M., Monari, M., Pandolfo, L., Pettinari, C. & Pombeiro, A. J. L. (2007). *Inorg. Chem.* **46**, 221–230. 10.1021/ic061595n 17198431

[bb7] Forward, J. M., Assefa, Z., Staples, R. J. & Fackler, J. P. Jr (1996). *Inorg. Chem.* **35**, 16–22. 10.1021/ic950560c 11666157

[bb8] Frost, B. J., Bautista, C. M., Huang, R. C. & Shearer, J. (2006). *Inorg. Chem.* **45**, 3481–3483. 10.1021/ic060322p 16634574

[bb9] Karabach, Y. Y., Kirillov, A. M., da Silva, M. F. C. G., Kopylovich, M. N. & Pombeiro, A. J. L. (2006). *Cryst. Growth Des.* **6**, 2200–2203.

[bb10] Kirillov, A. M., Karabach, Y. Y., Haukka, M., Guedes da Silva, M. F. C., Sanchiz, J., Kopylovich, M. N. & Pombeiro, A. J. L. (2008). *Inorg. Chem.* **47**, 162–175. 10.1021/ic701669x 18069826

[bb11] Kirillov, A. M., Smoleński, P., Guedes da Silva, M. F. C. & Pombeiro, A. J. L. (2007). *Eur. J. Inorg. Chem.* pp. 2686–2692.

[bb12] Lidrissi, C., Romerosa, A., Saoud, M., Serrano-Ruiz, M., Gonsalvi, L. & Peruzzini, M. (2005). *Angew. Chem. Int. Ed.* **44**, 2568–2572. 10.1002/anie.200462790 15782373

[bb13] Macrae, C. F., Edgington, P. R., McCabe, P., Pidcock, E., Shields, G. P., Taylor, R., Towler, M. & van de Streek, J. (2006). *J. Appl. Cryst.* **39**, 453–457.

[bb14] Mohr, F., Falvello, L. R. & Laguna, M. (2006). *Eur. J. Inorg. Chem.* pp. 3152–3154.

[bb15] Phillips, A. D., Gonsalvi, L., Romerosa, A., Vizza, F. & Peruzzini, M. (2004). *Coord. Chem. Rev.* **248**, 955–993.

[bb16] Sheldrick, G. M. (2003). *SADABS* Bruker AXS Inc., Madison, Wisconsin, USA.

[bb17] Sheldrick, G. M. (2008). *Acta Cryst.* A**64**, 112–122. 10.1107/S0108767307043930 18156677

[bb18] Spek, A. L. (2003). *J. Appl. Cryst.* **36**, 7–13.

